# Microglial Cells as a Link between Cannabinoids and the Immune Hypothesis of Psychiatric Disorders

**DOI:** 10.3389/fneur.2016.00005

**Published:** 2016-01-28

**Authors:** Sabrina F. Lisboa, Felipe V. Gomes, Francisco S. Guimaraes, Alline C. Campos

**Affiliations:** ^1^Department of Pharmacology, Medical School of Ribeirão Preto, University of São Paulo, Ribeirão Preto, Brazil; ^2^Center of Interdisciplinary Research on Applied Neurosciences (NAPNA), University of São Paulo, Ribeirão Preto, Brazil; ^3^Department of Neuroscience, University of Pittsburgh, Pittsburgh, PA, USA

**Keywords:** microglia, glia, cannabinoids, anxiety, depression, schizophrenia

## Abstract

Psychiatric disorders are one of the leading causes of disability worldwide. Although several therapeutic options are available, the exact mechanisms responsible for the genesis of these disorders remain to be fully elucidated. In the last decade, a body of evidence has supported the involvement of the immune system in the pathophysiology of these conditions. Microglial cells play a significant role in maintaining brain homeostasis and surveillance. Dysregulation of microglial functions has been associated with several psychiatric conditions. Cannabinoids regulate the brain–immune axis and inhibit microglial cell activation. Here, we summarized evidence supporting the hypothesis that microglial cells could be a target for cannabinoid influence on psychiatric disorders, such as anxiety, depression, schizophrenia, and stress-related disorders.

## Introduction

### Microglial Cells and Psychiatric Disorders

Over the last 20 years, both the innate and adaptive components of the immune system have been associated with the development of psychiatric disorders, such as depression (1) and schizophrenia (2). However, the mechanisms involved in this association are not altogether clear. Although a full review of these mechanisms would be out of the scope of this mini-review, recent evidence indicates that microglial cells could be important players in this complex puzzle and future targets for the treatment of these disorders (3).

Microglial cells are macrophage-like cells involved in immune surveillance of the central nervous system (CNS) and are a major source of inflammatory mediators in the brain (4). They originate from primitive myeloid progenitors before embryonic day 8 and from infiltrating myeloid cells during embryonic and postnatal development (5, 6). Microglia also contributes to CNS homeostasis and plasticity by removing redundant synapses and eliminating dying neurons; modulating neurotransmitter release and neurogenesis; and producing neurotrophic factors (7, 8).

During processes that challenge the brain milieu microglial cells proliferate and change their morphology from surveillance (ramified form) to executive and phagocytic state (amoeboid form, activated microglia) (9). Similar to peripheral macrophages, microglial cells assume at least two distinct states of polarization: M1, a profile that secretes proinflammatory cytokines, and M2, a pro-resolution state (4). The activated microglia releases proinflammatory mediators that, along with its phagocytic activity, may lead to brain damage and contribute to the development of psychiatric disorders (4).

### Immune System, Microglia, Anxiety, and Stress-Related Disorders

Stressful experiences such as social defeat activate long-lasting peripheral and central immune response (10–12) and induce microglial activation, myelopoiesis in the bone marrow and spleen, infiltration of monocytes into the brain and neuroinflammation (12–14).

In humans, posttraumatic stress disorder (PTSD) patients present increased peripheral levels of cytokines, in basal and inflammatory conditions (15, 16). Also, although no longer classified as an anxiety disorder, alterations in the immune system of patients with obsessive-compulsive disorder have also been reported (17, 18).

The activation and morphological changes of microglial cells associated with neuroinflammatory states have been recently found to depend on changes induced by stress, including the engagement of glucocorticoids and β-adrenergic receptors (19).

Pharmacological strategies to suppress microglial activity support the involvement of these cells in the development of disease- or stress-induced neuroinflammation and behavioral alterations (20–22). Minocycline is a tetracycline-derived antibiotic with central anti-inflammatory properties that readily crosses the blood–brain barrier (23, 24). It attenuates microglial activation, neuroinflammation, synaptic plasticity, neurogenesis, and behavioral changes in animal models of stress-related disorders (19, 22, 25–27) and after systemic lipopolysaccharides (LPSs) administration (28, 29). The mechanisms of minocycline anti-inflammatory effects are not clear, but may involve facilitation of endocannabinoid (eCB) signaling, since they can be prevented by CB1 or CB2 receptor antagonists (30). However, its effects in patients with anxiety disorders are still unknown.

Propranolol, a β-adrenergic receptor antagonist, also attenuates anxiety-like behavior, stress-induced brain proinflammatory profile (including infiltration of peripheral macrophages into the brain and microglial activation) (31, 32), and the increase in bone marrow monocytes progenitors (33). These effects could be due to an inhibitory effect on stress-induced peripheral immune system activation (12). Anti-inflammatory effects have also been described for antidepressant drugs after clinical and preclinical studies (34–36).

Overall, these results suggest that modulation of microglial proinflammatory profile, either centrally or by interference with peripheral sympathetic activity, could induce anxiolytic and antistress effects.

### Immune System, Microglia, and Depression

Patients with mood disorders exhibit an activated inflammatory status (37–39), characterized by increases in the number of circulating lymphocytes and macrophages and proinflammatory cytokines (40). Treatment of inflammatory conditions with interferon-α induces depressive symptoms and decreases serotonin levels in the prefrontal cortex of patients (41). These effects could be related to central activation of the enzyme indoleamine 2,3-dioxygenase (IDO) (42, 43). Proinflammatory cytokines are proposed to activate IDO that, by interfering with tryptophan/kynurenine metabolism, decreases serotonin levels and facilitates the production of quinolinic acid, an *N*-methyl-d-aspartate (NMDA) receptor agonist (40, 44). Microglial and astrocyte cells control IDO activity. Moreover, activated microglia and infiltrated macrophages have a high capacity for synthesizing quinolinic acid (45). Victims of suicide with the history of affective disorder have increased density of activated phagocytes in the ventral prefrontal white matter (46) and upregulation of the gene IBA1, associated with phenotypic changes in microglia, and MCP-1, a chemokine responsible for attracting monocytes, in the dorsal anterior cingulate (47, 48). Besides the increased number of activated microglial cells are reported in the hippocampus of bipolar patients (49).

Antidepressant drugs are reported to inhibit IL-6 (50, 51) and TNF-α production (52). Antidepressants inhibit LPS-stimulated microglia (36). Moreover, fluoxetine prevents IκB-α degradation and NF-κB nuclear translocation (53), while lithium decreases LPS-induced microglial activation through the PI3K/Akt/FoxO1 signaling pathway (54). Corroborating these findings, studies suggest that anti-inflammatory drugs as add-on therapy to antidepressant medication may boost depression treatment (55–57).

Stressful experiences are highly associated with predisposition for both depression episodes and immune dysfunction (58, 59). Stress activates microglia in the prefrontal cortex, amygdala, and hippocampus of mice (60) and impairs synaptic plasticity by reducing neuronal activity and decreasing dendritic spine density (61). The high levels of proinflammatory cytokines secreted by microglia downregulate neurotrophic factors, intracellular growth pathways, and neurogenesis (61, 62), in which mechanisms believe to be downregulated in depressive states.

### Immune System, Microglia, and Schizophrenia

Increased expression of inflammatory markers in blood and brain tissues (63–65) and changes in genes that control the expression of immune system components have been described in schizophrenia patients (66). Prenatal exposure to inflammatory agents increases the risk for schizophrenia development (67) and meta-analyses indicate the potential use of anti-inflammatory drugs as adjunct treatment in schizophrenia (68).

Postmortem brains of schizophrenia patients present activation and increased cellular density of microglia (69–71). The latter finding has been confirmed by positron emission tomography studies using *in vivo* markers of activated microglia (72–74). Additionally, elevated microglial activity is also observed in people at ultra high risk of psychosis and is associated with symptom severity, suggesting a link between microglial activation and the risk of psychosis (74). Increased microglial activation is also observed in animal models of schizophrenia (75, 76). Although it is unclear how changes in microglial activity result in schizophrenia symptoms, there seems to be an association between microglial activation and negative and cognitive symptoms (77, 78). In line with this proposition, minocycline improved negative symptoms and cognitive function as an add-on treatment in schizophrenia patients (77, 79, 80). Antipsychotic-like effects of minocycline have also been observed in preclinical studies (81, 82). Together, these results suggest that inhibition of microglial activation may improve schizophrenia symptoms.

## Cannabinoids as Immunomodulators in the Central Nervous System

The eCB system was initially described in the late 1980s after the identification of specific receptors (83). It now comprises the cannabinoid receptor types 1 (CB1) and 2 (CB2), their endogenous ligands anandamide (AEA) and 2-arachidonoylglycerol (2-AG), and the enzymes responsible for their synthesis and degradation (84–86).

In the CNS, eCBs modulate synaptic function and act as a homeostatic mechanism on HPA axis (87). During stressful or threatening situations, eCBs favor HPA axis activation through the amygdala. Glucocorticoids, by enhancing the production of eCB, modulate glutamatergic and GABAergic neurotransmission *via* CB1 receptors (87). These receptors are highly expressed in presynaptic terminals and their activation suppresses the release of several neurotransmitters, such as glutamate, GABA, and serotonin (88). CB1 is also expressed in astrocytes (89), oligodendrocytes (90), and neural precursor cells, which also expresses CB2 receptors (91). In addition to CB1, CB2 receptors are constitutively expressed in microglia cells (92) and its expression increases in inflammatory conditions (93). These receptors have been proposed as key regulators of the immune functions, including microglial activation (94–96). They are overexpressed during neurodegenerative diseases, such as Alzheimer’s disease and multiple sclerosis, conditions in which activated microglia is observed (97). Recently, Mecha et al. (98) demonstrate that the eCB system, by activating CB2 receptors, not only influences the migration, proliferation, and release of proinflammatory mediators of microglial cells but also affects their phagocytic function and drive these glial cells to the M2 state.

2-Arachidonoylglycerol can protect neurons exposed to harmful insults by acting as an endogenous inhibitor of cyclooxygenase-2 (COX-2) (99), whereas AEA inhibits TNF-α-induced NF-κB activation by direct inhibition of the IκB kinase (100). Pharmacological inhibition of AEA hydrolysis reduces microglial activation, nitric oxide levels, and the production of inflammatory mediators (101). Under pathological conditions, microglia cells produce large amounts of eCBs, which could facilitate an anti-inflammatory phenotype of microglia (92). Enzymes controlling eCB tone also plays an important neuroprotective role during neuroinflammatory process (97). Supporting the involvement of eCBs in immune modulation, the neuroprotective effect and inhibition of microglial activation induced by minocycline were prevented by CB1 and CB2 receptor antagonists in a rodent model of traumatic brain injury (30).

Exogenous cannabinoids can also modulate microglia activation (97, 102, 103). They reduce the binding of transcription factors to CRE and NF-κB in immune cells (104) and inhibit cytokine and chemokine production (105). WIN55,212-2, a mixed CB1/CB2 receptor agonist, reduced brain mRNA expression of proinflammatory cytokines, such as TNF-α and IL-6, in a viral model of multiple sclerosis (106) and in the Alzheimer’s disease model of Aβ amyloid injection (107, 108). Moreover, WIN55,212-2 also decreased the number of activated microglia related to Aβ administration (107) or the aging process in rats (102).

### Cannabinoids, Microglia, and Anxiety Disorder

Overexpression of CB1 and CB2 receptors, or their acute pharmacological activation, promotes anxiolytic-like effects (109, 110), whereas their genetic deletion or pharmacological blockade causes opposite results (111, 112). These receptors also attenuate the increased proinflammatory profile observed in the frontal cortex after subchronic stress in mice (113, 114), reducing microglial activation and proliferation (95, 115–117).

Cannabinoids could also attenuate anxiety by modulating the release of IL-1ra, the endogenous antagonist of IL-1β, by glial cells in response to glutamate (118), and by interfering with the HPA axis (119). In the latter case, glucocorticoids modulate microglial activation induced by stressors (120, 121) and suppress hippocampal and amygdala eCB signaling (122).

### Cannabinoids, Microglia, and Depression

Lipopolysaccharide induces “sickness behavior” in rodents, a syndrome that shows some similarity with depressive symptoms and depends on prolonged cytokines release and microglial activation (123, 124). Accordingly, using LPS as inflammatory stimulus, cannabinoids reduced the number of circulating lymphocyte, corticosterone levels (125), and the release of IL-1β, TNF-α, and iNOS expression *in vitro* (126). Moreover, the long-lasting behavioral deficits induced by LPS are prevented by the administration of THC (127) or cannabidiol (CBD) (108). As discussed above, in addition to interfere with HPA axis (119), cannabinoids can directly decrease microglial activation and attenuate stress-induced neuroinflammatory states (108, 125, 126).

Although the specific contribution of CB1 and CB2 receptors for the aforementioned anti-inflammatory effects is still unclear, the neuroprotective effects of CB2 agonists are associated with a decrease in the number of activated microglial cells (107). *In vitro* studies indicate that these receptors, located at microglial cells, facilitate the production of anti-inflammatory mediators (128). Considering the pieces of evidence suggesting that depression could be a “microglial disease,” these results point to CB2 receptors located at this cells as possible targets for future antidepressant treatments.

### Cannabinoids, Microglia, and Schizophrenia

Adolescent cannabis exposure represents a risk factor for developing schizophrenia later in life (129). Besides the long-lasting changes in neuronal activity induced by adolescent cannabinoid exposure (76, 130), increased microglial activation in the prefrontal cortex (131) and hippocampus of adult rats have also been observed (132). Moreover, ibudilast (AV411), a non-selective phosphodiesterase inhibitor that suppresses glial cell activation (133), prevents the development of behavioral changes induced by adolescent THC exposure (131).

Unlike THC, CBD is a phytocannabinoid devoid of psychotomimetic activity that present antipsychotic activity (134). The mechanism of action involved in this effect is not entirely understood. However, the anti-inflammatory and neuroprotective effects of this drug (135) may contribute to its beneficial effects in schizophrenia. Repeated treatment with CBD-attenuated behavioral deficits and the percentage of Iba-1-positive microglial cells with a reactive phenotype in the medial prefrontal cortex and dorsal hippocampus of mice chronically treated with the NMDA receptor antagonist MK-801 (136). CBD treatment also attenuated the decreased number of GABAergic parvalbumin-positive cells in the medial prefrontal cortex, which could, by reducing inhibitory tonus in this region, facilitate glutamate release and lead to microglial activation (137). Interestingly, schizophrenia patients with a higher inflammatory state had more deficits in GABAergic neuron-related mRNAs, including GAD67 and parvalbumin (138).

Regarding the eCB system, whereas higher levels of 2-AG have been observed in the prefrontal cortex, hippocampus, and cerebellum of schizophrenia patients, AEA levels are lower (139). Moreover, increased AEA levels in the cerebrospinal fluid correlate negatively with psychotic symptoms (140) and the antipsychotic effect of CBD was associated with increased AEA serum levels. This latter effect likely reflects CBD inhibition of the FAAH enzyme, responsible for AEA degradation (141). Increases in eCBs may contribute to defense mechanisms through accumulation of anti-inflammatory microglia phenotype (92). Thus, the pharmacological inhibition of eCB hydrolysis may be a useful approach in the schizophrenia treatment.

As aforementioned, CB2 receptors are expressed on microglia and its expression is strongly upregulated when these cells are activated. Schizophrenia has been associated with single nucleotide polymorphisms in the CB2 receptor gene that reduce its expression and functionality (142). Decreased expression of CB2 receptors in isolated peripheral blood mononuclear cells is found during first-episode psychosis (143). However, no study has evaluated changes on CB2 receptor expression in microglia cells in the brain of schizophrenia patients. In rodents, pharmacological or genetic CB2 receptor blockade increases susceptibility for developing schizophrenia-like symptoms (111, 142). Additionally, a CB2 receptor agonist reversed sensorimotor gating deficits in mice induced by MK-801 (144). However, the involvement of microglial CB2 receptors in these effects is unknown.

## Conclusion

**Figure 1:**
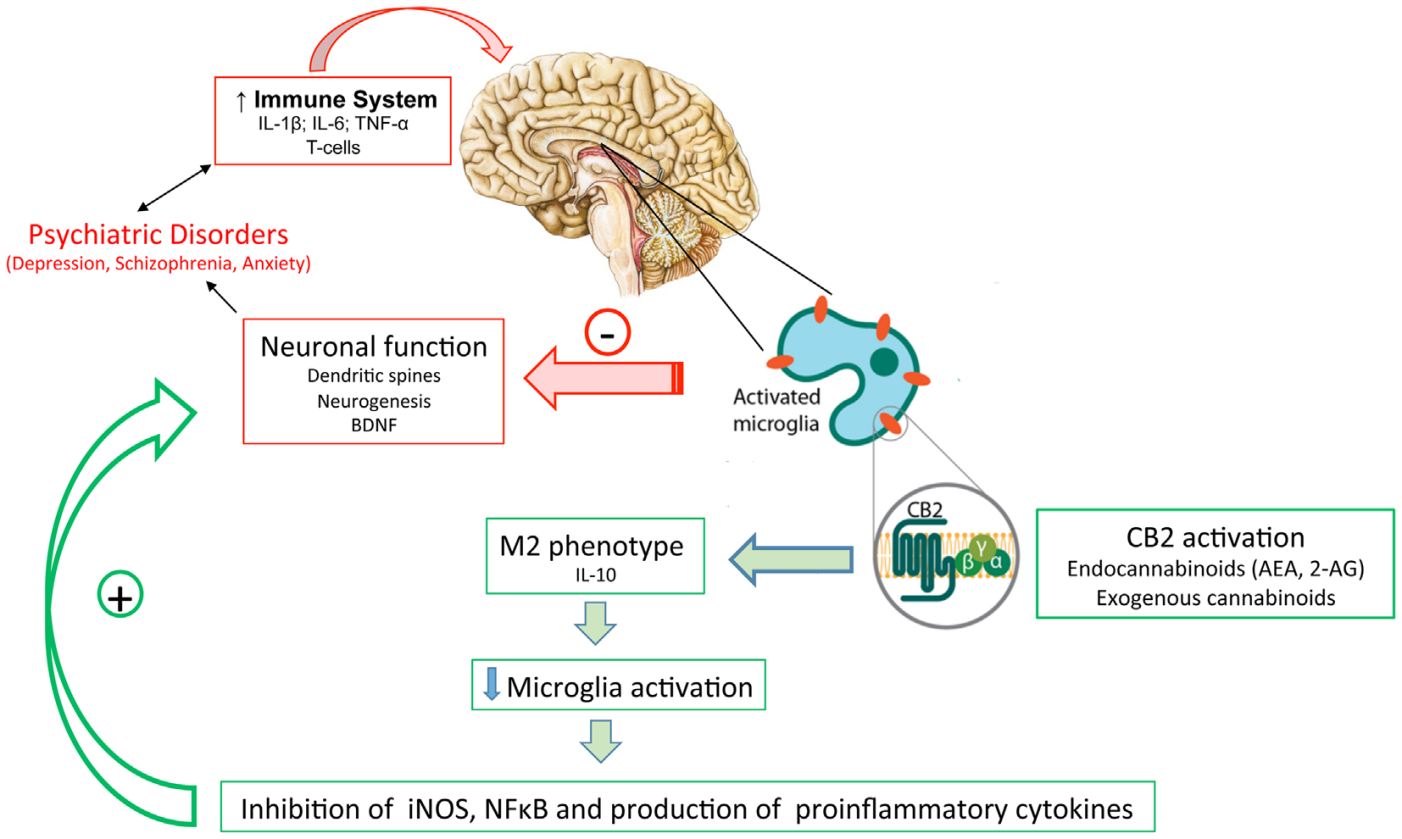
**The inflammatory status observed in patients with psychiatric disorders might lead to activated microglia and neuronal/synaptic damage**. Activation of cannabinoids receptors (mainly CB2 in activated microglia) by (endo)cannabinoids would drive microglia to a M2 (pro-resolution) state, decreasing the production of proinflammatory mediators and protecting neurons from damage.

A large body of evidence supports the involvement of neuroinflammatory mechanisms, including microglial activation, in the pathophysiology of psychiatric disorders. Drugs that interfere with these mechanisms, such as cannabinoids, could be a novel and important new pathway for the treatment of these disorders (Figure 1). Despite these pieces of evidence, few studies have yet directly investigated if interference with microglial cell activation is essential for the therapeutic effects of cannabinoids in psychiatric disorders. Additional studies, therefore, are needed to test this hypothesis.

## Author Contributions

All authors have contributed in the writing part of this mini-review.

## Conflict of Interest Statement

The authors declare that the research was conducted in the absence of any commercial or financial relationships that could be construed as a potential conflict of interest.

## References

[1] Haapakoski R Ebmeier KP Alenius H Kivimaki M. Innate and adaptive immunity in the development of depression: an update on current knowledge and technological advances. Prog Neuropsychopharmacol Biol Psychiatry (2015) 66:63–72. 10.1016/j.pnpbp.2015.11.012 26631274 PMC4736094

[2] Watkins CC Andrews SR Clinical studies of neuroinflammatory mechanisms in schizophrenia. Schizophr Res (2015). 10.1016/j.schres.2015.07.018 26235751

[3] Yirmiya R Rimmerman N Reshef R. Depression as a microglial disease. Trends Neurosci (2015) 38:637–58. 10.1016/j.tins.2015.08.001 26442697

[4] Prinz M Priller J. Microglia and brain macrophages in the molecular age: from origin to neuropsychiatric disease. Nat Rev Neurosci (2014) 15:300–12. 10.1038/nrn3722 24713688

[5] Alliot F Godin I Pessac B. Microglia derive from progenitors, originating from the yolk sac, and which proliferate in the brain. Brain Res Dev Brain Res (1999) 117:145–52. 10.1016/S0165-3806(99)00113-3 10567732

[6] Ginhoux F Greter M Leboeuf M Nandi S See P Gokhan S Fate mapping analysis reveals that adult microglia derive from primitive macrophages. Science (2010) 330:841–5. 10.1126/science.1194637 20966214 PMC3719181

[7] Ziv Y Avidan H Pluchino S Martino G Schwartz M. Synergy between immune cells and adult neural stem/progenitor cells promotes functional recovery from spinal cord injury. Proc Natl Acad Sci U S A (2006) 103:13174–9. 10.1073/pnas.0603747103 16938843 PMC1559772

[8] Beumer W Gibney SM Drexhage RC Pont-Lezica L Doorduin J Klein HC The immune theory of psychiatric diseases: a key role for activated microglia and circulating monocytes. J Leukoc Biol (2012) 92:959–75. 10.1189/jlb.0212100 22875882

[9] Ransohoff RM Cardona AE. The myeloid cells of the central nervous system parenchyma. Nature (2010) 468:253–62. 10.1038/nature09615 21068834

[10] Avitsur R Powell N Padgett DA Sheridan JF Social interactions, stress, and immunity. Immunol Allergy Clin North Am (2009) 29:285–93. 10.1016/j.iac.2009.02.006 19389582

[11] Wohleb ES McKim DB Shea DT Powell ND Tarr AJ Sheridan JF Re-establishment of anxiety in stress-sensitized mice is caused by monocyte trafficking from the spleen to the brain. Biol Psychiatry (2014) 75:970–81. 10.1016/j.biopsych.2013.11.029 24439304 PMC4084643

[12] Reader BF Jarrett BL McKim DB Wohleb ES Godbout JP Sheridan JF. Peripheral and central effects of repeated social defeat stress: monocyte trafficking, microglial activation, and anxiety. Neuroscience (2015) 289:429–42. 10.1016/j.neuroscience.2015.01.001 25596319 PMC4536813

[13] Wohleb ES McKim DB Sheridan JF Godbout JP Monocyte trafficking to the brain with stress and inflammation: a novel axis of immune-to-brain communication that influences mood and behavior. Front Neurosci (2015) 8:447 10.3389/fnins.2014.00447 25653581 PMC4300916

[14] McKim DB Patterson JM Wohleb ES Jarrett BL Reader BF Godbout JP Sympathetic release of splenic monocytes promotes recurring anxiety following repeated social defeat. Biol Psychiatry (2015). 10.1016/j.biopsych.2015.07.010 26281717 PMC4728074

[15] Rohleder N Nater UM Wolf JM Ehlert U Kirschbaum C. Psychosocial stress-induced activation of salivary alpha-amylase: an indicator of sympathetic activity? Ann N Y Acad Sci (2004) 1032:258–63. 10.1196/annals.1314.033 15677423

[16] Lindqvist D Wolkowitz OM Mellon S Yehuda R Flory JD Henn-Haase C Proinflammatory milieu in combat-related PTSD is independent of depression and early life stress. Brain Behav Immun (2014) 42:81–8. 10.1016/j.bbi.2014.06.003 24929195

[17] da Rocha FF Correa H Teixeira AL. Obsessive-compulsive disorder and immunology: a review. Prog Neuropsychopharmacol Biol Psychiatry (2008) 32:1139–46. 10.1016/j.pnpbp.2007.12.026 18262706

[18] Cappi C Muniz RK Sampaio AS Cordeiro Q Brentani H Palacios SA Association study between functional polymorphisms in the TNF-alpha gene and obsessive-compulsive disorder. Arq Neuropsiquiatr (2012) 70:87–90. 10.1590/S0004-282X2012000200003 22311210 PMC4479271

[19] Yuan TF Hou G Zhao Y Arias-Carrion O. Commentary: the effects of psychological stress on microglial cells in the brain. CNS Neurol Disord Drug Targets (2015) 14:304–8. 10.2174/1871527314666150123122851 25613648

[20] Kim HS Suh YH. Minocycline and neurodegenerative diseases. Behav Brain Res (2009) 196:168–79. 10.1016/j.bbr.2008.09.040 18977395

[21] Hinwood M Morandini J Day TA Walker FR. Evidence that microglia mediate the neurobiological effects of chronic psychological stress on the medial prefrontal cortex. Cereb Cortex (2012) 22:1442–54. 10.1093/cercor/bhr229 21878486

[22] Levkovitz Y Fenchel D Kaplan Z Zohar J Cohen H. Early post-stressor intervention with minocycline, a second-generation tetracycline, attenuates post-traumatic stress response in an animal model of PTSD. Eur Neuropsychopharmacol (2015) 25:124–32. 10.1016/j.euroneuro.2014.11.012 25487770

[23] Colovic M Caccia S. Liquid chromatographic determination of minocycline in brain-to-plasma distribution studies in the rat. J Chromatogr B Analyt Technol Biomed Life Sci (2003) 791:337–43. 10.1016/S1570-0232(03)00247-2 12798193

[24] Dean OM Data-Franco J Giorlando F Berk M. Minocycline: therapeutic potential in psychiatry. CNS Drugs (2012) 26:391–401. 10.2165/11632000-000000000-00000 22486246

[25] Ekdahl CT Claasen JH Bonde S Kokaia Z Lindvall O. Inflammation is detrimental for neurogenesis in adult brain. Proc Natl Acad Sci U S A (2003) 100:13632–7. 10.1073/pnas.2234031100 14581618 PMC263865

[26] Pabreja K Dua K Sharma S Padi SS Kulkarni SK. Minocycline attenuates the development of diabetic neuropathic pain: possible anti-inflammatory and anti-oxidant mechanisms. Eur J Pharmacol (2011) 661:15–21. 10.1016/j.ejphar.2011.04.014 21536024

[27] Taylor AM Castonguay A Taylor AJ Murphy NP Ghogha A Cook C Microglia disrupt mesolimbic reward circuitry in chronic pain. J Neurosci (2015) 35:8442–50. 10.1523/JNEUROSCI.4036-14.2015 26041913 PMC4452552

[28] Tomás-Camardiel M Rite I Herrera AJ de Pablos RM Cano J Machado A Minocycline reduces the lipopolysaccharide-induced inflammatory reaction, peroxynitrite-mediated nitration of proteins, disruption of the blood-brain barrier, and damage in the nigral dopaminergic system. Neurobiol Dis (2004) 16:190–201. 10.1016/j.nbd.2004.01.010 15207276

[29] Henry CJ Huang Y Wynne A Hanke M Himler J Bailey MT Minocycline attenuates lipopolysaccharide (LPS)-induced neuroinflammation, sickness behavior, and anhedonia. J Neuroinflammation (2008) 5:15. 10.1186/1742-2094-5-15 18477398 PMC2412862

[30] Lopez-Rodriguez AB Siopi E Finn DP Marchand-Leroux C Garcia-Segura LM Jafarian-Tehrani M CB1 and CB2 cannabinoid receptor antagonists prevent minocycline-induced neuroprotection following traumatic brain injury in mice. Cereb Cortex (2015) 25:35–45. 10.1093/cercor/bht202 23960212

[31] Wohleb ES Hanke ML Corona AW Powell ND Stiner LM Bailey MT Beta-adrenergic receptor antagonism prevents anxiety-like behavior and microglial reactivity induced by repeated social defeat. J Neurosci (2011) 31:6277–88. 10.1523/JNEUROSCI.0450-11.2011 21525267 PMC3160240

[32] Hanke ML Powell ND Stiner LM Bailey MT Sheridan JF. Beta adrenergic blockade decreases the immunomodulatory effects of social disruption stress. Brain Behav Immun (2012) 26:1150–9. 10.1016/j.bbi.2012.07.011 22841997 PMC3506115

[33] Powell ND Sloan EK Bailey MT Arevalo JM Miller GE Chen E Social stress up-regulates inflammatory gene expression in the leukocyte transcriptome via beta-adrenergic induction of myelopoiesis. Proc Natl Acad Sci U S A (2013) 110:16574–9. 10.1073/pnas.1310655110 24062448 PMC3799381

[34] Sutcigil L Oktenli C Musabak U Bozkurt A Cansever A Uzun O Pro- and anti-inflammatory cytokine balance in major depression: effect of sertraline therapy. Clin Dev Immunol (2007) 2007:76396. 10.1155/2007/76396 18317531 PMC2248234

[35] Norden DM Devine R Bicer S Jing R Reiser PJ Wold LE Fluoxetine prevents the development of depressive-like behavior in a mouse model of cancer related fatigue. Physiol Behav (2015) 140:230–5. 10.1016/j.physbeh.2014.12.045 25554480 PMC4298482

[36] Ramirez K Shea DT McKim DB Reader BF Sheridan JF. Imipramine attenuates neuroinflammatory signaling and reverses stress-induced social avoidance. Brain Behav Immun (2015) 46:212–20. 10.1016/j.bbi.2015.01.016 25701613 PMC4414808

[37] Schlatter J Ortuno F Cervera-Enguix S. Differences in interleukins’ patterns between dysthymia and major depression. Eur Psychiatry (2001) 16:317–9. 10.1016/S0924-9338(01)00585-5 11514136

[38] Tuglu C Kara SH Caliyurt O Vardar E Abay E. Increased serum tumor necrosis factor-alpha levels and treatment response in major depressive disorder. Psychopharmacology (Berl) (2003) 170:429–33. 10.1007/s00213-003-1566-z 12955291

[39] Grassi-Oliveira R Bauer ME Pezzi JC Teixeira AL Brietzke E. Interleukin-6 and verbal memory in recurrent major depressive disorder. Neuro Endocrinol Lett (2011) 32:540–4. 21876502

[40] Muller N. Immunology of major depression. Neuroimmunomodulation (2014) 21:123–30. 10.1159/000356540 24557045

[41] McNamara RK Lotrich FE. Elevated immune-inflammatory signaling in mood disorders: a new therapeutic target? Expert Rev Neurother (2012) 12:1143–61. 10.1586/ern.12.98 23039393 PMC3535180

[42] Raison CL Dantzer R Kelley KW Lawson MA Woolwine BJ Vogt G CSF concentrations of brain tryptophan and kynurenines during immune stimulation with IFN-alpha: relationship to CNS immune responses and depression. Mol Psychiatry (2010) 15:393–403. 10.1038/mp.2009.116 19918244 PMC2844942

[43] Wichers MC Koek GH Robaeys G Verkerk R Scharpe S Maes M. IDO and interferon-alpha-induced depressive symptoms: a shift in hypothesis from tryptophan depletion to neurotoxicity. Mol Psychiatry (2005) 10:538–44. 10.1038/sj.mp.4001600 15494706

[44] Kim JS Schmid-Burgk W Claus D Kornhuber HH. Effects of amitriptyline on serum glutamate and free tryptophan in rats. Arch Psychiatr Nervenkr (1970) (1982) 232:391–4. 10.1007/BF00345595 6133513

[45] Heyes MP Achim CL Wiley CA Major EO Saito K Markey SP. Human microglia convert l-tryptophan into the neurotoxin quinolinic acid. Biochem J (1996) 320(Pt 2):595–7. 10.1042/bj3200595 8973572 PMC1217971

[46] Schnieder TP Trencevska I Rosoklija G Stankov A Mann JJ Smiley J Microglia of prefrontal white matter in suicide. J Neuropathol Exp Neurol (2014) 73:880–90. 10.1097/NEN.0000000000000107 25101704 PMC4141011

[47] Steiner J Walter M Gos T Guillemin GJ Bernstein HG Sarnyai Z Severe depression is associated with increased microglial quinolinic acid in subregions of the anterior cingulate gyrus: evidence for an immune-modulated glutamatergic neurotransmission? J Neuroinflammation (2011) 8:94. 10.1186/1742-2094-8-94 21831269 PMC3177898

[48] Torres-Platas SG Cruceanu C Chen GG Turecki G Mechawar N. Evidence for increased microglial priming and macrophage recruitment in the dorsal anterior cingulate white matter of depressed suicides. Brain Behav Immun (2014) 42:50–9. 10.1016/j.bbi.2014.05.007 24858659

[49] Haarman BC Riemersma-Van der Lek RF de Groot JC Ruhe HG Klein HC Zandstra TE Neuroinflammation in bipolar disorder – A [(11)C]-(R)-PK11195 positron emission tomography study. Brain Behav Immun (2014) 40:219–25. 10.1016/j.bbi.2014.03.016 24703991

[50] Xia Z DePierre JW Nassberger L. Tricyclic antidepressants inhibit IL-6, IL-1 beta and TNF-alpha release in human blood monocytes and IL-2 and interferon-gamma in T cells. Immunopharmacology (1996) 34:27–37. 10.1016/0162-3109(96)00111-7 8880223

[51] Basterzi AD Aydemir C Kisa C Aksaray S Tuzer V Yazici K IL-6 levels decrease with SSRI treatment in patients with major depression. Hum Psychopharmacol (2005) 20:473–6. 10.1002/hup.717 16158446

[52] Schiepers OJ Wichers MC Maes M. Cytokines and major depression. Prog Neuropsychopharmacol Biol Psychiatry (2005) 29:201–17. 10.1016/j.pnpbp.2004.11.003 15694227

[53] Liu D Wang Z Liu S Wang F Zhao S Hao A. Anti-inflammatory effects of fluoxetine in lipopolysaccharide(LPS)-stimulated microglial cells. Neuropharmacology (2011) 61:592–9. 10.1016/j.neuropharm.2011.04.033 21575647

[54] Dong H Zhang X Dai X Lu S Gui B Jin W Lithium ameliorates lipopolysaccharide-induced microglial activation via inhibition of toll-like receptor 4 expression by activating the PI3K/Akt/FoxO1 pathway. J Neuroinflammation (2014) 11:140. 10.1186/s12974-014-0140-4 25115727 PMC4149204

[55] Nery FG Monkul ES Hatch JP Fonseca M Zunta-Soares GB Frey BN Celecoxib as an adjunct in the treatment of depressive or mixed episodes of bipolar disorder: a double-blind, randomized, placebo-controlled study. Hum Psychopharmacol (2008) 23:87–94. 10.1002/hup.912 18172906

[56] Akhondzadeh S Jafari S Raisi F Nasehi AA Ghoreishi A Salehi B Clinical trial of adjunctive celecoxib treatment in patients with major depression: a double blind and placebo controlled trial. Depress Anxiety (2009) 26:607–61. 10.1002/da.20589 19496103

[57] Guo JY Li CY Ruan YP Sun M Qi XL Zhao BS Chronic treatment with celecoxib reverses chronic unpredictable stress-induced depressive-like behavior via reducing cyclooxygenase-2 expression in rat brain. Eur J Pharmacol (2009) 612:54–60. 10.1016/j.ejphar.2009.03.076 19356723

[58] Bidzinska E [Premorbid personality characteristics in patients with affective disorders]. Psychiatr Pol (1984) 18:313–8. 6536016

[59] Dowlati Y Herrmann N Swardfager W Liu H Sham L Reim EK A meta-analysis of cytokines in major depression. Biol Psychiatry (2010) 67:446–57. 10.1016/j.biopsych.2009.09.033 20015486

[60] Delpech JC Madore C Nadjar A Joffre C Wohleb ES Laye S. Microglia in neuronal plasticity: influence of stress. Neuropharmacology (2015) 96:19–28. 10.1016/j.neuropharm.2014.12.034 25582288

[61] Kreisel T Frank MG Licht T Reshef R Ben-Menachem-Zidon O Baratta MV Dynamic microglial alterations underlie stress-induced depressive-like behavior and suppressed neurogenesis. Mol Psychiatry (2014) 19:699–709. 10.1038/mp.2013.155 24342992

[62] Campos AC Vaz GN Saito VM Teixeira AL. Further evidence for the role of interferon-gamma on anxiety- and depressive-like behaviors: involvement of hippocampal neurogenesis and NGF production. Neurosci Lett (2014) 578:100–5. 10.1016/j.neulet.2014.06.039 24993299

[63] Kirkpatrick B Miller BJ Inflammation and schizophrenia. Schizophr Bull (2013) 39:1174–9. 10.1093/schbul/sbt141 24072812 PMC3796097

[64] Fillman SG Cloonan N Catts VS Miller LC Wong J McCrossin T Increased inflammatory markers identified in the dorsolateral prefrontal cortex of individuals with schizophrenia. Mol Psychiatry (2013) 18:206–14. 10.1038/mp.2012.110 22869038

[65] Volk DW Chitrapu A Edelson JR Roman KM Moroco AE Lewis DA Molecular mechanisms and timing of cortical immune activation in schizophrenia. Am J Psychiatry (2015) 172:1112–21. 10.1176/appi.ajp.2015.15010019 26133963 PMC5063256

[66] Schizophrenia Working Group of the Psychiatric Genomics Consortium. Biological insights from 108 schizophrenia-associated genetic loci. Nature (2014) 511:42–427. 10.1038/nature13595 25056061 PMC4112379

[67] Clarke MC Tanskanen A Huttunen M Whittaker JC Cannon M. Evidence for an interaction between familial liability and prenatal exposure to infection in the causation of schizophrenia. Am J Psychiatry (2009) 166:1025–30. 10.1176/appi.ajp.2009.08010031 19487391

[68] Sommer IE van Westrhenen R Begemann MJ de Witte LD Leucht S Kahn RS. Efficacy of anti-inflammatory agents to improve symptoms in patients with schizophrenia: an update. Schizophr Bull (2014) 40:181–91. 10.1093/schbul/sbt139 24106335 PMC3885306

[69] Bayer TA Buslei R Havas L Falkai P. Evidence for activation of microglia in patients with psychiatric illnesses. Neurosci Lett (1999) 271:126–8. 10.1016/S0304-3940(99)00545-5 10477118

[70] Radewicz K Garey LJ Gentleman SM Reynolds R. Increase in HLA-DR immunoreactive microglia in frontal and temporal cortex of chronic schizophrenics. J Neuropathol Exp Neurol (2000) 59:137–50. 10.1093/jnen/59.2.137 10749103

[71] Steiner J Bielau H Bernstein HG Bogerts B Wunderlich MT. Increased cerebrospinal fluid and serum levels of S100B in first-onset schizophrenia are not related to a degenerative release of glial fibrillar acidic protein, myelin basic protein and neurone-specific enolase from glia or neurones. J Neurol Neurosurg Psychiatry (2006) 77:1284–7. 10.1136/jnnp.2006.093427 17043297 PMC2077376

[72] van Berckel BN Bossong MG Boellaard R Kloet R Schuitemaker A Caspers E Microglia activation in recent-onset schizophrenia: a quantitative (R)-[11C]PK11195 positron emission tomography study. Biol Psychiatry (2008) 64:820–2. 10.1016/j.biopsych.2008.04.025 18534557

[73] Doorduin J de Vries EF Willemsen AT de Groot JC Dierckx RA Klein HC. Neuroinflammation in schizophrenia-related psychosis: a PET study. J Nucl Med (2009) 50:1801–7. 10.2967/jnumed.109.066647 19837763

[74] Bloomfield PS Selvaraj S Veronese M Rizzo G Bertoldo A Owen DR Microglial activity in people at ultra high risk of psychosis and in schizophrenia: an [C]PBR28 PET Brain Imaging Study. Am J Psychiatry (2016) 173:44–52. 10.1176/appi.ajp.2015.14101358 26472628 PMC4821370

[75] Ribeiro BM do Carmo MR Freire RS Rocha NF Borella VC de Menezes AT Evidences for a progressive microglial activation and increase in iNOS expression in rats submitted to a neurodevelopmental model of schizophrenia: reversal by clozapine. Schizophr Res (2013) 151:12–9. 10.1016/j.schres.2013.10.040 24257517

[76] Gomes FV Guimaraes FS Grace AA Effects of pubertal cannabinoid administration on attentional set-shifting and dopaminergic hyper-responsivity in a developmental disruption model of schizophrenia. Int J Neuropsychopharmacol (2015) 18:1–10. 10.1093/ijnp/pyu018 PMC4368886 25522381

[77] Levkovitz Y Mendlovich S Riwkes S Braw Y Levkovitch-Verbin H Gal G A double-blind, randomized study of minocycline for the treatment of negative and cognitive symptoms in early-phase schizophrenia. J Clin Psychiatry (2010) 71:138–49. 10.4088/JCP.08m04666yel 19895780

[78] Ribeiro-Santos A Lucio Teixeira A Salgado JV. Evidence for an immune role on cognition in schizophrenia: a systematic review. Curr Neuropharmacol (2014) 12:273–80. 10.2174/1570159X1203140511160832 24851091 PMC4023457

[79] Chaudhry IB Hallak J Husain N Minhas F Stirling J Richardson P Minocycline benefits negative symptoms in early schizophrenia: a randomised double-blind placebo-controlled clinical trial in patients on standard treatment. J Psychopharmacol (2012) 26:1185–93. 10.1177/0269881112444941 22526685

[80] Chaves C Marque CR Maia-de-Oliveira JP Wichert-Ana L Ferrari TB Santos AC Effects of minocycline add-on treatment on brain morphometry and cerebral perfusion in recent-onset schizophrenia. Schizophr Res (2015) 161:439–45. 10.1016/j.schres.2014.11.031 25497439

[81] Fujita Y Ishima T Kunitachi S Hagiwara H Zhang L Iyo M Phencyclidine-induced cognitive deficits in mice are improved by subsequent subchronic administration of the antibiotic drug minocycline. Prog Neuropsychopharmacol Biol Psychiatry (2008) 32:336–9. 10.1016/j.pnpbp.2007.08.031 17884273

[82] Mattei D Djodari-Irani A Hadar R Pelz A de Cossio LF Goetz T Minocycline rescues decrease in neurogenesis, increase in microglia cytokines and deficits in sensorimotor gating in an animal model of schizophrenia. Brain Behav Immun (2014) 38:175–84. 10.1016/j.bbi.2014.01.019 24509090

[83] Devane WA Dysarz FA III Johnson MR Melvin LS Howlett AC. Determination and characterization of a cannabinoid receptor in rat brain. Mol Pharmacol (1988) 34:605–13. 2848184

[84] Devane WA Hanus L Breuer A Pertwee RG Stevenson LA Griffin G Isolation and structure of a brain constituent that binds to the cannabinoid receptor. Science (1992) 258:1946–9. 10.1126/science.1470919 1470919

[85] Mechoulam R Ben-Shabat S Hanus L Ligumsky M Kaminski NE Schatz AR Identification of an endogenous 2-monoglyceride, present in canine gut, that binds to cannabinoid receptors. Biochem Pharmacol (1995) 50:83–90. 10.1016/0006-2952(95)00109-D 7605349

[86] Katona I Freund TF. Endocannabinoid signaling as a synaptic circuit breaker in neurological disease. Nat Med (2008) 14:923–30. 10.1038/nm.f.1869 18776886

[87] Hillard CJ. Stress regulates endocannabinoid-CB1 receptor signaling. Semin Immunol (2014) 26:380–8. 10.1016/j.smim.2014.04.001 24882055 PMC4247817

[88] Moreira FA Aguiar DC Campos AC Lisboa SF Terzian AL Resstel LB Antiaversive effects of cannabinoids: is the periaqueductal gray involved? Neural Plast (2009) 2009:625469. 10.1155/2009/625469 19096514 PMC2593468

[89] Navarrete M Araque A. Endocannabinoids potentiate synaptic transmission through stimulation of astrocytes. Neuron (2010) 68:113–26. 10.1016/j.neuron.2010.08.043 20920795

[90] Mato S Victoria Sanchez-Gomez M Matute C. Cannabidiol induces intracellular calcium elevation and cytotoxicity in oligodendrocytes. Glia (2010) 58:1739–47. 10.1002/glia.21044 20645411

[91] Maccarrone M Guzman M Mackie K Doherty P Harkany T. Programming of neural cells by (endo)cannabinoids: from physiological rules to emerging therapies. Nat Rev Neurosci (2014) 15:786–801. 10.1038/nrn3846 25409697 PMC4765324

[92] Stella N. Endocannabinoid signaling in microglial cells. Neuropharmacology (2009) 56(Suppl 1):244–53. 10.1016/j.neuropharm.2008.07.037 18722389 PMC2654419

[93] Cabral GA Marciano-Cabral F. Cannabinoid receptors in microglia of the central nervous system: immune functional relevance. J Leukoc Biol (2005) 78:1192–7. 10.1189/jlb.0405216 16204639

[94] Correa F Mestre L Docagne F Guaza C. Activation of cannabinoid CB2 receptor negatively regulates IL-12p40 production in murine macrophages: role of IL-10 and ERK1/2 kinase signaling. Br J Pharmacol (2005) 145:441–8. 10.1038/sj.bjp.0706215 15821753 PMC1576166

[95] Ehrhart J Obregon D Mori T Hou H Sun N Bai Y Stimulation of cannabinoid receptor 2 (CB2) suppresses microglial activation. J Neuroinflammation (2005) 2:29. 10.1186/1742-2094-2-29 16343349 PMC1352348

[96] Ashton JC Cannabinoids for the treatment of inflammation. Curr Opin Investig Drugs (2007) 8:373–84. 17520866

[97] Bisogno T Di Marzo V. Cannabinoid receptors and endocannabinoids: role in neuroinflammatory and neurodegenerative disorders. CNS Neurol Disord Drug Targets (2010) 9:564–73. 10.2174/187152710793361568 20632970

[98] Mecha M Feliu A Carrillo-Salinas FJ Rueda-Zubiaurre A Ortega-Gutierrez S de Sola RG Endocannabinoids drive the acquisition of an alternative phenotype in microglia. Brain Behav Immun (2015) 49:233–45. 10.1016/j.bbi.2015.06.002 26086345

[99] Du H Chen X Zhang J Chen C Inhibition of COX-2 expression by endocannabinoid 2-arachidonoylglycerol is mediated via PPAR-gamma. Br J Pharmacol (2011) 163:1533–49. 10.1111/j.1476-5381.2011.01444.x 21501147 PMC3165961

[100] Sancho R Calzado MA Di Marzo V Appendino G Munoz E. Anandamide inhibits nuclear factor-kappaB activation through a cannabinoid receptor-independent pathway. Mol Pharmacol (2003) 63:429–38. 10.1124/mol.63.2.429 12527815

[101] Murphy N Cowley TR Blau CW Dempsey CN Noonan J Gowran A The fatty acid amide hydrolase inhibitor URB597 exerts anti-inflammatory effects in hippocampus of aged rats and restores an age-related deficit in long-term potentiation. J Neuroinflammation (2012) 9:79. 10.1186/1742-2094-9-79 22537429 PMC3409037

[102] Marchalant Y Brothers HM Norman GJ Karelina K DeVries AC Wenk GL. Cannabinoids attenuate the effects of aging upon neuroinflammation and neurogenesis. Neurobiol Dis (2009) 34:300–7. 10.1016/j.nbd.2009.01.014 19385063

[103] Henry RJ Kerr DM Finn DP Roche M. For whom the endocannabinoid tolls: modulation of innate immune function and implications for psychiatric disorders. Prog Neuropsychopharmacol Biol Psychiatry (2016) 64:167–80. 10.1016/j.pnpbp.2015.03.006 25794989

[104] Herring AC Kaminski NE. Cannabinol-mediated inhibition of nuclear factor-kappaB, cAMP response element-binding protein, and interleukin-2 secretion by activated thymocytes. J Pharmacol Exp Ther (1999) 291:1156–63. 10565837

[105] Rossi S Motta C Musella A Centonze D. The interplay between inflammatory cytokines and the endocannabinoid system in the regulation of synaptic transmission. Neuropharmacology (2015) 96:105–12. 10.1016/j.neuropharm.2014.09.022 25268960

[106] Arevalo-Martin A Garcia-Ovejero D Sierra-Palomares Y Paniagua-Torija B Gonzalez-Gil I Ortega-Gutierrez S Early endogenous activation of CB1 and CB2 receptors after spinal cord injury is a protective response involved in spontaneous recovery. PLoS One (2012) 7:e49057. 10.1371/journal.pone.0049057 23152849 PMC3496738

[107] Ramírez BG Blazquez C Gomez del Pulgar T Guzman M de Ceballos ML. Prevention of Alzheimer’s disease pathology by cannabinoids: neuroprotection mediated by blockade of microglial activation. J Neurosci (2005) 25:1904–13. 10.1523/JNEUROSCI.4540-04.2005 15728830 PMC6726060

[108] Martín-Moreno AM Reigada D Ramirez BG Mechoulam R Innamorato N Cuadrado A Cannabidiol and other cannabinoids reduce microglial activation in vitro and in vivo: relevance to Alzheimer’s disease. Mol Pharmacol (2011) 79:964–73. 10.1124/mol.111.071290 21350020 PMC3102548

[109] Valenzano KJ Tafesse L Lee G Harrison JE Boulet JM Gottshall SL Pharmacological and pharmacokinetic characterization of the cannabinoid receptor 2 agonist, GW405833, utilizing rodent models of acute and chronic pain, anxiety, ataxia and catalepsy. Neuropharmacology (2005) 48:658–72. 10.1016/j.neuropharm.2004.12.008 15814101

[110] García-Gutiérrez MS Manzanares J. Overexpression of CB2 cannabinoid receptors decreased vulnerability to anxiety and impaired anxiolytic action of alprazolam in mice. J Psychopharmacol (2011) 25:111–20. 10.1177/0269881110379507 20837564

[111] Ortega-Alvaro A Aracil-Fernandez A Garcia-Gutierrez MS Navarrete F Manzanares J. Deletion of CB2 cannabinoid receptor induces schizophrenia-related behaviors in mice. Neuropsychopharmacology (2011) 36:1489–504. 10.1038/npp.2011.34 21430651 PMC3096817

[112] García-Gutiérrez MS Garcia-Bueno B Zoppi S Leza JC Manzanares J. Chronic blockade of cannabinoid CB2 receptors induces anxiolytic-like actions associated with alterations in GABA(A) receptors. Br J Pharmacol (2012) 165:951–64. 10.1111/j.1476-5381.2011.01625.x 21838753 PMC3312491

[113] Zoppi S Perez Nievas BG Madrigal JL Manzanares J Leza JC Garcia-Bueno B. Regulatory role of cannabinoid receptor 1 in stress-induced excitotoxicity and neuroinflammation. Neuropsychopharmacology (2011) 36:805–18. 10.1038/npp.2010.214 21150911 PMC3055736

[114] Zoppi S Madrigal JL Caso JR Garcia-Gutierrez MS Manzanares J Leza JC Regulatory role of the cannabinoid CB2 receptor in stress-induced neuroinflammation in mice. Br J Pharmacol (2014) 171:2814–26. 10.1111/bph.12607 24467609 PMC4243857

[115] Walter L Franklin A Witting A Wade C Xie Y Kunos G Nonpsychotropic cannabinoid receptors regulate microglial cell migration. J Neurosci (2003) 23:1398–405. 12598628 10.1523/JNEUROSCI.23-04-01398.2003 PMC6742252

[116] Fernández-Ruiz J Pazos MR Garcia-Arencibia M Sagredo O Ramos JA. Role of CB2 receptors in neuroprotective effects of cannabinoids. Mol Cell Endocrinol (2008) 286:S91–6. 10.1016/j.mce.2008.01.001 18291574

[117] Romero-Sandoval EA Horvath R Landry RP DeLeo JA. Cannabinoid receptor type 2 activation induces a microglial anti-inflammatory phenotype and reduces migration via MKP induction and ERK dephosphorylation. Mol Pain (2009) 5:25. 10.1186/1744-8069-5-25 19476641 PMC2704199

[118] Molina-Holgado F Pinteaux E Moore JD Molina-Holgado E Guaza C Gibson RM Endogenous interleukin-1 receptor antagonist mediates anti-inflammatory and neuroprotective actions of cannabinoids in neurons and glia. J Neurosci (2003) 23:6470–4. 12878687 10.1523/JNEUROSCI.23-16-06470.2003 PMC6740626

[119] Akirav I. Cannabinoids and glucocorticoids modulate emotional memory after stress. Neurosci Biobehav Rev (2013) 37:2554–63. 10.1016/j.neubiorev.2013.08.002 23954749

[120] Frank MG Thompson BM Watkins LR Maier SF. Glucocorticoids mediate stress-induced priming of microglial pro-inflammatory responses. Brain Behav Immun (2012) 26:337–45. 10.1016/j.bbi.2011.10.005 22041296 PMC5652300

[121] Carrillo-de Sauvage MA Maatouk L Arnoux I Pasco M Sanz Diez A Delahaye M Potent and multiple regulatory actions of microglial glucocorticoid receptors during CNS inflammation. Cell Death Differ (2013) 20:1546–57. 10.1038/cdd.2013.108 24013726 PMC3792430

[122] Bowles NP Hill MN Bhagat SM Karatsoreos IN Hillard CJ McEwen BS Chronic, noninvasive glucocorticoid administration suppresses limbic endocannabinoid signaling in mice. Neuroscience (2012) 204:83–9. 10.1016/j.neuroscience.2011.08.048 21939741 PMC3697830

[123] Perry VH. The influence of systemic inflammation on inflammation in the brain: implications for chronic neurodegenerative disease. Brain Behav Immun (2004) 18:407–13. 10.1016/j.bbi.2004.01.004 15265532

[124] Dantzer R O’Connor JC Freund GG Johnson RW Kelley KW. From inflammation to sickness and depression: when the immune system subjugates the brain. Nat Rev Neurosci (2008) 9:46–56. 10.1038/nrn2297 18073775 PMC2919277

[125] Roche M Diamond M Kelly JP Finn DP. In vivo modulation of LPS-induced alterations in brain and peripheral cytokines and HPA axis activity by cannabinoids. J Neuroimmunol (2006) 181:57–67. 10.1016/j.jneuroim.2006.08.001 17011047

[126] Ma L Jia J Liu X Bai F Wang Q Xiong L. Activation of murine microglial N9 cells is attenuated through cannabinoid receptor CB2 signaling. Biochem Biophys Res Commun (2015) 458:92–7. 10.1016/j.bbrc.2015.01.073 25637536

[127] Fishbein-Kaminietsky M Gafni M Sarne Y. Ultralow doses of cannabinoid drugs protect the mouse brain from inflammation-induced cognitive damage. J Neurosci Res (2014) 92:1669–77. 10.1002/jnr.23452 25042014

[128] Carrier EJ Kearn CS Barkmeier AJ Breese NM Yang W Nithipatikom K Cultured rat microglial cells synthesize the endocannabinoid 2-arachidonylglycerol, which increases proliferation via a CB2 receptor-dependent mechanism. Mol Pharmacol (2004) 65:999–1007. 10.1124/mol.65.4.999 15044630

[129] Silins E Horwood LJ Patton GC Fergusson DM Olsson CA Hutchinson DM Young adult sequelae of adolescent cannabis use: an integrative analysis. Lancet Psychiatry (2014) 1:286–93. 10.1016/S2215-0366(14)70307-4 26360862

[130] Cass DK Flores-Barrera E Thomases DR Vital WF Caballero A Tseng KY. CB1 cannabinoid receptor stimulation during adolescence impairs the maturation of GABA function in the adult rat prefrontal cortex. Mol Psychiatry (2014) 19:536–43. 10.1038/mp.2014.14 24589887 PMC3999247

[131] Zamberletti E Gabaglio M Prini P Rubino T Parolaro D. Cortical neuroinflammation contributes to long-term cognitive dysfunctions following adolescent delta-9-tetrahydrocannabinol treatment in female rats. Eur Neuropsychopharmacol (2015) 25:2404–15. 10.1016/j.euroneuro.2015.09.021 26499171

[132] Lopez-Rodriguez AB Llorente-Berzal A Garcia-Segura LM Viveros MP. Sex-dependent long-term effects of adolescent exposure to THC and/or MDMA on neuroinflammation and serotoninergic and cannabinoid systems in rats. Br J Pharmacol (2014) 171:1435–47. 10.1111/bph.12519 24236988 PMC3954483

[133] Mizuno T Kurotani T Komatsu Y Kawanokuchi J Kato H Mitsuma N Neuroprotective role of phosphodiesterase inhibitor ibudilast on neuronal cell death induced by activated microglia. Neuropharmacology (2004) 46:404–11. 10.1016/j.neuropharm.2003.09.009 14975696

[134] Campos AC Moreira FA Gomes FV Del Bel EA Guimaraes FS. Multiple mechanisms involved in the large-spectrum therapeutic potential of cannabidiol in psychiatric disorders. Philos Trans R Soc Lond B Biol Sci (2012) 367:3364–78. 10.1098/rstb.2011.0389 23108553 PMC3481531

[135] Izzo AA Borrelli F Capasso R Di Marzo V Mechoulam R. Non-psychotropic plant cannabinoids: new therapeutic opportunities from an ancient herb. Trends Pharmacol Sci (2009) 30:515–27. 10.1016/j.tips.2009.07.006 19729208

[136] Gomes FV Llorente R Del Bel EA Viveros MP Lopez-Gallardo M Guimaraes FS Decreased glial reactivity could be involved in the antipsychotic-like effect of cannabidiol. Schizophr Res (2015) 164:155–63. 10.1016/j.schres.2015.01.015 25680767

[137] Gomes FV Issy AC Ferreira FR Viveros MP Del Bel EA Guimaraes FS Cannabidiol attenuates sensorimotor gating disruption and molecular changes induced by chronic antagonism of NMDA receptors in mice. Int J Neuropsychopharmacol (2015) 18:1–10. 10.1093/ijnp/pyu041 PMC4376539 25618402

[138] Fillman SG Cloonan N Miller LC Weickert CS Markers of inflammation in the prefrontal cortex of individuals with schizophrenia. Mol Psychiatry (2013) 18:133 10.1038/mp.2012.110 23344565

[139] Muguruza C Lehtonen M Aaltonen N Morentin B Meana JJ Callado LF. Quantification of endocannabinoids in postmortem brain of schizophrenic subjects. Schizophr Res (2013) 148:145–50. 10.1016/j.schres.2013.06.013 23800614

[140] Giuffrida A Leweke FM Gerth CW Schreiber D Koethe D Faulhaber J Cerebrospinal anandamide levels are elevated in acute schizophrenia and are inversely correlated with psychotic symptoms. Neuropsychopharmacology (2004) 29:2108–14. 10.1038/sj.npp.1300558 15354183

[141] Leweke FM Piomelli D Pahlisch F Muhl D Gerth CW Hoyer C Cannabidiol enhances anandamide signaling and alleviates psychotic symptoms of schizophrenia. Transl Psychiatry (2012) 2:e94. 10.1038/tp.2012.15 22832859 PMC3316151

[142] Ishiguro H Horiuchi Y Ishikawa M Koga M Imai K Suzuki Y Brain cannabinoid CB2 receptor in schizophrenia. Biol Psychiatry (2010) 67:974–82. 10.1016/j.biopsych.2009.09.024 19931854

[143] Bioque M García-Bueno B Macdowell KS Meseguer A Saiz PA Parellada M; FLAMM-PEPs study—Centro de Investigacion Biomedica en Red de Salud Mental. Peripheral endocannabinoid system dysregulation in first-episode psychosis. Neuropsychopharmacology (2013) 38:2568–77. 10.1038/npp.2013.165 23822951 PMC3828529

[144] Khella R Short JL Malone DT. CB2 receptor agonism reverses MK-801-induced disruptions of prepulse inhibition in mice. Psychopharmacology (Berl) (2014) 231:3071–87. 10.1007/s00213-014-3481-x 24705902

